# Reference values for the alcohol biomarker phosphatidylethanol (PEth) in the Belgian population: Insights from a nationwide microsampling study

**DOI:** 10.1016/j.dadr.2026.100451

**Published:** 2026-05-23

**Authors:** Kevin Vandenbroucke, Katleen Van Uytfanghe, Liesl Heughebaert, Nicolas Berger, Evy De Boosere, Christophe P. Stove

**Affiliations:** a Laboratory of Toxicology, Department of Bioanalysis, Faculty of Pharmaceutical Sciences, Ghent University, Ghent, Belgium; b Department of Epidemiology and Public Health, Scientific Institute of Public Health (Sciensano), Brussels, Belgium; c Department of Forensic Pathology, Ghent University Hospital, Ghent, Belgium

**Keywords:** PEth, Alcohol use, VAMS, AUDIT-C, LC-MS/MS

## Abstract

**Background:**

Phosphatidylethanol 16:0/18:1 (PEth) is a well-established direct biomarker for monitoring recent and chronic alcohol consumption. However, population-based surveys assessing PEth in both people who drink alcohol and people who do not drink alcohol remain limited, despite their value for contextualizing forensic and clinical results.

**Methods:**

PEth was quantified in samples collected during the 2022–2023 Belgian National Food Consumption Survey (n = 487) using capillary volumetric absorptive microsampling (VAMS) and an ISO17025-accredited liquid chromatography-tandem mass spectrometry method (10–2000 ng/mL). Participants also completed an AUDIT-C questionnaire; relationships with PEth were evaluated using Spearman correlation and Cohen’s kappa. Weighted data were used to derive estimates for the general Belgian population and compared with two presumed higher-consumption subgroups.

**Results:**

PEth concentrations in the general Belgian population ranged from <10 to 1400 ng/mL. Overall, 35.7% had concentrations between 20 and 200 ng/mL and 10.7% exceeded 200 ng/mL. PEth correlated strongly with AUDIT-C scores, with Spearman’s rank correlation coefficients of 0.61 in males and 0.64 in females. Agreement between PEth and AUDIT-C for identifying hazardous drinking was moderate in males and substantial in females (Cohen’s kappa: 0.55 and 0.63, respectively). The median PEth concentration in the general population was 17 ng/mL (95% CI: 15–18), compared with 32 ng/mL in a non-abstinent adult population and 122 ng/mL in individuals enrolled in a driver’s license regranting program.

**Conclusion:**

These findings provide population-based PEth reference data for Belgium and demonstrate clear differences in PEth distributions between general and higher-exposure populations, supporting improved contextual interpretation of forensic and clinical PEth results.

## Highlights

• Nationwide survey assessing PEth via volumetric absorptive microsampling (VAMS).
• Median PEth levels in the general adult Belgian population are below 20 ng/mL
• PEth levels indicated chronic excessive drinking in 11% of the adult population.
• Strong correlation between PEth and self-reported alcohol use (AUDIT-C).
• Higher PEth in non-abstinent and forensic groups than in the general population.

## 1 Introduction

Assessing alcohol consumption, either for forensic or clinical purposes, has been a recognized procedure for over decades. Whereas recent consumption is determined via ethanol measurement, long-term alcohol use can be assessed using several tools (Andresen-Streichert et al., 2018). A straightforward and inexpensive tool is self-reporting via the World Health Organization's 10-item Alcohol Use Disorders Identification Test (AUDIT) or the shorter 3-item AUDIT-C questionnaire to identify hazardous and harmful drinking (Babor et al., 2001, Bush et al., 1998). A more objective method to estimate alcohol (mis)use, not prone to (intentional) inaccuracies or recall bias is the quantification of specific biomarkers in biological matrices (Afshar et al., 2022). Both indirect biomarkers, e.g. liver enzymes and carbohydrate-deficient transferrin, whose activity or formation is influenced by alcohol intake, and direct biomarkers can be measured (Alladio et al., 2017, Nanau and Neuman, 2015). The latter show superior sensitivity and specificity as they are only produced when ethanol is present, and have ethanol incorporated into their chemical structure (Andresen-Streichert et al., 2018, Jones, 2019, Stewart et al., 2014). Among the direct biomarkers, phosphatidylethanol (PEth), a group of abnormal phospholipids, has received increasing interest.

At the erythrocyte cell membrane, PEth is generated shortly after alcohol consumption by the enzyme phospholipase D, through the reaction of ethanol with various phospholipids (Alling et al., 1983, Varga and Alling, 2002). The most abundant product is PEth 16:0/18:1, which is commonly quantified in whole blood samples using liquid chromatography-tandem mass spectrometry (LC-MS/MS) (Nguyen et al., 2018, White et al., 2022, Zheng et al., 2011). PEth is detectable in blood for extended periods due to its slow degradation (half-life ~7.9 days), rendering it suitable for monitoring both short- and long-term alcohol use (Javors et al., 2016, Van Uytfanghe et al., 2022b). Currently, PEth has applications in forensic and clinical settings, including diagnosing alcohol use disorders, detecting foetal alcohol syndrome, and longitudinal monitoring of alcohol use in specific populations such as people enrolled in driver’s license regranting programs (Luginbühl et al., 2022b, Van Uytfanghe et al., 2022a, Wurst et al., 2015). As defined by the 2022 Basel Consensus, threshold PEth concentrations of 20 and 200 ng/mL are commonly used in alcohol abstinence monitoring and forensic practice. Values above these thresholds are respectively compatible with alcohol consumption or strongly suggestive of chronic excessive alcohol consumption (Luginbühl et al., 2022a). The latter is defined by the World Health Organization as an average intake of ≥ 60 g of pure alcohol per day in the last year (World Health Organization, 2024).

As PEth is measured in whole blood, it can also be quantified via dried blood microsampling (Beck et al., 2018, Kummer et al., 2016a, Kummer et al., 2016b, Luginbühl et al., 2021), as routinely done at the Laboratory of Toxicology at Ghent University. Using only 10 µL of capillary blood wicked up by an absorbent polymeric tip (volumetric absorptive microsampling (VAMS)), PEth 16:0/18:1 is quantified via LC-MS/MS (Van Uytfanghe et al., 2021a). The lab developed a population-based model to assess abstinence following consecutive positive PEth results, based on serial PEth measurements in 687 participants (non-abstainers) who self-reported one month of abstinence (Van Uytfanghe et al., 2022b). This study yielded insight into PEth’s half-life and provided objective evidence supporting the 20 ng/mL cut-off to conclude compatibility with abstinence or low alcohol consumption for about 1 month (Van Uytfanghe and Stove, 2023). However, as the cohort only included individuals with drinking habits, general population reference values remained undetermined, and Belgian population studies were lacking.

Since 2004, Belgium’s national public health institute, Sciensano, has assessed dietary habits and nutritional status through the nationwide Belgian Food Consumption Survey (BFCS). In the BFCS, trained interviewers collect detailed dietary questionnaires from individuals, which are a representative sample of the Belgian population. In addition to obtaining food consumption data via questionnaires, the latest BFCS also included an ‘add-on’, in which dried blood microsamples (dried blood spots and VAMS samples) were collected from the participants. These dried blood microsamples were used to determine two vitamins (thiamine and vitamin D) and PEth (Heughebaert et al., 2026, Lebacq et al., 2024a). The primary aim of this study was to assess the distribution of PEth concentrations in the general adult Belgian population. Secondary aims included evaluating the relationship between PEth concentrations and self-reported alcohol consumption using AUDIT-C, and contextualizing the obtained population-based PEth data through comparison with two in-house datasets representing populations with presumed higher alcohol exposure. Together, these data may support improved interpretation of PEth concentrations in forensic and clinical settings.

## 2 Materials and methods

### 2.1 Study design

This study was conducted in the context of the BFCS, which is a cross-sectional epidemiologic study assessing nutrition and eating habits in Belgian individuals aged ≥ 3 years. Only participants aged 18 or older were included for PEth-based assessment of alcohol intake. Adults were randomly selected from the Belgian National Register using a multistage stratified clustered sampling design, incorporating stratification by province, followed by selection of municipalities and individuals, to ensure a representative sample of the adult population. Selected individuals received an invitation and were visited by a trained interviewer on two non-consecutive days. Participants provided informed consent for study participation, biological sample analysis, and linkage of results to questionnaire data. All data and biological samples were anonymized to ensure privacy. The study was conducted in accordance with the Declaration of Helsinki and received approval of the Ethics Committee of Ghent University Hospital (BC-10616 AM01).

### 2.2 Sampling procedure

Assisted self-sampling using VAMS was performed at the participant’s home under supervision of a trained interviewer. The aim was to collect up to three good quality capillary blood samples on 10 µL VAMS devices (Mitra®; Trajan Scientific, Melbourne, Australia). For detailed information on the study workflow, we refer to Heughebaert et al. (2026) Briefly, following disinfection of the skin with an isopropanol wipe, a finger prick was performed whereafter the first drop of blood was wiped off, and the next drops were absorbed by the device’s polymeric tip. After sampling, VAMS devices were air-dried for at least 2 h before being put in a sealed zip-lock plastic bag with silica desiccant, which was sent to the lab via regular mail. Upon arrival, the samples underwent a visual quality inspection to verify sample integrity. Samples were excluded from analysis if they did not meet the pre-specified quality criteria, including underfilled samples (visible white areas of the polymeric tip), overfilled samples (dark to black discoloration of the tip and/or visible blood contamination on the plastic holder), or devices with inadequate storage during transportation (sent without zip-lock bag and/or silica desiccant) (Boffel et al., 2024).

VAMS samples were collected with a dual purpose, i.e. for thiamine measurement and for PEth determination, with the first good quality sample of a given participant being used for thiamine determination and the second good quality sample being used to measure PEth (Heughebaert et al., 2026). After applying the inclusion criteria, a total of 487 samples were retained for PEth analysis.

### 2.3 Participant information

During interviewer visits, participants completed questionnaires to collect information on (i) sociodemographics; (ii) alcohol drinking behaviour using the AUDIT-C questionnaire; (iii) dietary intake, evaluated using a 24-hour dietary recall on two non-consecutive days including a food frequency questionnaire, and (iv) health status, physical activity, sedentary behaviour etc. For data collection on sensitive topics such as mental health, a self-administered questionnaire was used. Participants’ weight, height and waist circumference were also objectively measured by the interviewer (Lebacq et al., 2024b).

### 2.4 Variables

The primary outcome was the individual PEth concentration measured in VAMS samples, categorized according to the 2022 Basel Consensus as < 20 ng/mL (abstinence or low intake), 20–200 ng/mL (moderate intake), and > 200 ng/mL (chronic excessive consumption) (Luginbühl et al., 2022a). Covariates included sex (male, female), age category (18–39, 40–64, 65 +), highest household education level (secondary or lower, short-type higher education, long-type higher education), household type (single, single with children, couple, couple with children, other) and financial hardship, via asking “How easily can you make ends meet?” (very easily, easily, rather easily, rather difficultly, difficultly, very difficultly). In addition, the AUDIT-C score was evaluated to screen for hazardous alcohol consumption, defined as a score ≥ 4 for males or ≥ 3 for females.

### 2.5 PEth measurement

PEth (16:0/18:1) in VAMS samples was quantified using an ISO17025-accredited LC-MS/MS method (Van Uytfanghe et al., 2021b). The laboratory has also successfully participated in external quality control schemes for PEth analysis, including Equalis since 2018 and PEth.NET since 2020. Briefly, air-dried VAMS tips were extracted with 250 µL of solvent (2 mM ammonium acetate, 0.01% formic acid in water/isopropanol/formic acid, 2:8:0.2) and 60 µL of internal standard (PEth-d5, 25 ng/mL in methanol). After shaking (1400 rpm, 23°C, 60 min), 1 mL n-hexane was added for liquid-liquid extraction, followed by shaking and centrifugation (10 min, 10000*g*). The organic phase was dried under nitrogen (40°C), reconstituted in 50 µL injection solvent, and 5 µL was injected for LC-MS/MS analysis (quantifier *m/z* 701 → 255). Two configurations were used interchangeably, i.e. a Shimadzu Prominence LC (Shimadzu, Kyoto, Japan) coupled to a QTRAP® 5500 instrument (SCIEX, Framingham, MA, USA) and an Acquity UPLC® I class coupled to a Xevo TQ-S mass spectrometer (Waters, Milford, MA, USA). The lower limit of quantification (LLoQ) was 10 ng/mL and the calibration range was up to 2000 ng/mL.

### 2.6 Comparison to higher-exposure subgroups

To assess the suitability of the data as a reference for the general population, results were compared with two subgroups presumed to have higher alcohol consumption: (i) PEth concentrations from the first sampling point in the aforementioned study (Van Uytfanghe et al., 2022b), which included a non-abstinent adult population (NAAP), and (ii) independent PEth concentrations from individuals enrolled in a driver’s license regranting program (DLRP) within forensic medicine. All PEth data used were obtained in-house using the same measurement procedure and analyzed stratified by sex.

### 2.7 Statistical methods

As PEth is only present in PWDA, people who do not drink alcohol exhibit non-detectable levels (i.e. no chromatographic peak), while people who occasionally drink alcohol may have values < 10 ng/mL as well. To retain data below the LLoQ, the 'LLoQ/2' method was applied, substituting values < 10 ng/mL with 5 ng/mL (Keizer et al., 2015). To better represent the Belgian population, results were weighted using previously calculated weighting factors from the BFCS Survey 2022–2023 sample (Lebacq et al., 2024b). Differences between subgroups were considered very likely to reflect true population differences when the 95% confidence intervals (CI) of the calculated parameters did not overlap. Spearman’s rank correlation assessed the association between PEth concentrations and AUDIT-C scores, while Cohen’s kappa evaluated their agreement in identifying hazardous alcohol consumption. Descriptive data analysis was performed using IBM SPSS Statistics version 29 (IBM SPSS Statistics, Chicago, IL, USA), with data visualization using GraphPad Prism version 10.0.0 (GraphPad Software, Boston, MA, USA).

## 3 Results

### 3.1 Participant data

**Fig. 1:**
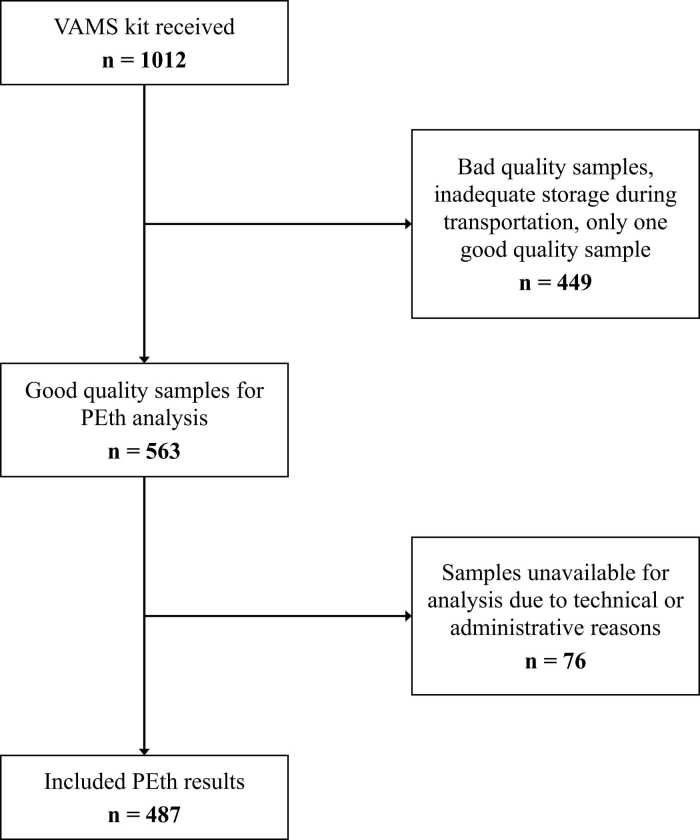
Flowchart illustrating inclusion and exclusion of volumetric absorptive microsampling (VAMS) samples for PEth determination. A total of 487 samples were retained for PEth analysis.

For detailed information regarding participation rate, sample quality and transport, we refer to Heughebaert et al. (2026). A total of 487 samples were available for PEth analysis with corresponding completed questionnaires (Fig. 1). The sample population was equally divided according to sex and age, with ages ranging from 18.7 to 93.4 years (median 55.7 years). The majority (~89.2%) of participants reported Belgian nationality. An overview of the sample and subgroup distributions is provided in Table 1.

**Table 1:** Demographic and study-related characteristics of the sample population, including subgroup distributions. Data are presented as counts and percentages (unweighted).

	Sample population
All participants	487
Sex	
Male	242 (49.7%)
Female	245 (50.3%)
Age	
18–39	151 (31.0%)
40–64	156 (32.0%)
65 and older	180 (37.0%)
Education level	
Secondary or lower	208 (42.7%)
Short-type higher education	140 (28.7%)
Long-type higher education	137 (28.1%)
Missing	2 (0.4%)
Household type	
Single	78 (16.0%)
Single with children	26 (5.3%)
Couple	167 (34.3%)
Couple with children	155 (31.8%)
Other	61 (12.5%)
Financial hardship	
Very easily	70 (14.4%)
Easily	176 (36.1%)
Rather easily	144 (29.6%)
Rather difficultly	60 (12.3%)
Difficultly	20 (4.1%)
Very easily	9 (1.8%)
Not specified	8 (1.6%)

**Table 2:** Weighted median and maximum PEth concentrations (ng/mL) and 95% confidence intervals for all subgroups.

	Median PEth conc. (95% CI)	Max PEth conc.
General population	17 (15, 18)	1400
Sex		
Male	34 (31, 37)	1216
Female	*<LLoQ <LLoQ*	1400
Age		
18–39	11 (11, 13)	430
40–64	16 (14, 20)	860
65 and older	20 (19, 26)	1400
Education level		
Secondary or lower	19 (15, 20)	1216
Short-type higher education	12 (11, 13)	618
Long-type higher education	19 (18, 23)	1400
Missing	<*LLoQ <LLoQ*	87
Household type		
Single	20 (15, 26)	1216
Single with children	24 (22, 34)	493
Couple	19 (16, 21)	1400
Couple with children	16 (14, 18)	705
Other	*<LLoQ <LLoQ*	719
Financial hardship		
Very easily	12 (11, 20)	618
Easily	17 (16, 17)	860
Rather easily	23 (18, 26)	1400
Rather difficultly	11 (<*LLoQ*, 13)	1215
Difficultly	14 (14, 38)	719
Very difficultly	<*LLoQ* (<*LLoQ*, 10)	682
Not specified	31 (28, 31)	210

### 3.2 Descriptive statistics

**Fig. 2:**
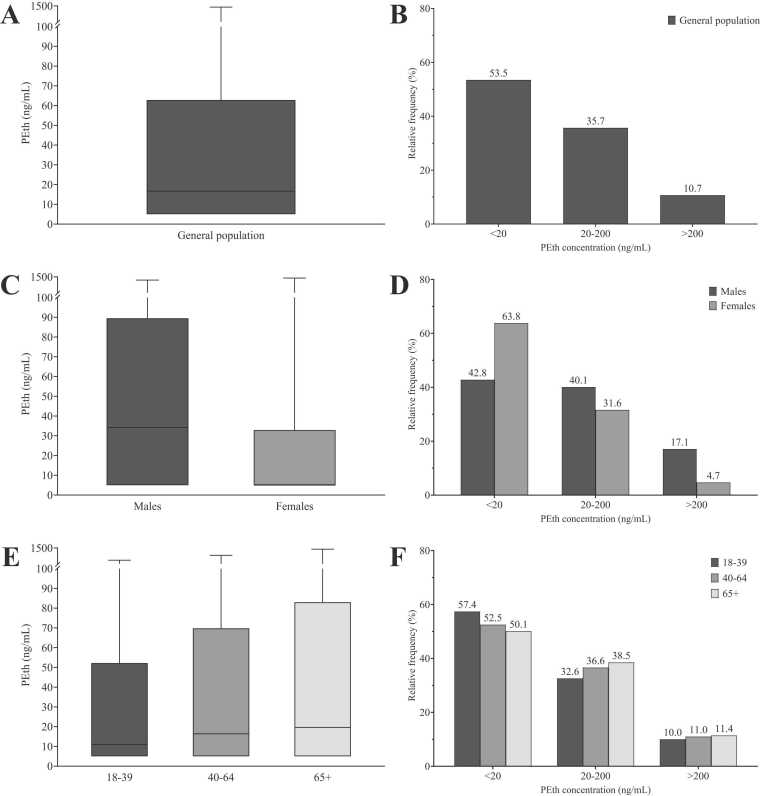
Boxplots showing PEth concentrations (ng/mL) in the general Belgian population (A), stratified by sex (C) and age (E). All concentrations <LLoQ of 10 ng/mL were substituted by the value of 5, whiskers indicating minimum-maximum range. Median concentrations: A = 17 ng/mL; C = 34 ng/mL (male) and <LLoQ (female); E = 11 ng/mL (18−39), 16 ng/mL (40−64), and 20 ng/mL (65 +). Bars represent the relative frequency of the general population (B), stratified by sex (D) and age (F) within three PEth categories (<20 ng/mL, 20–200 ng/mL, >200 ng/mL).

The weighted results yielded a PEth concentration range from <LLoQ to 1400 ng/mL, with a mean and median of 73 and 17 ng/mL, respectively. The data, stratified using the 20 and 200 ng/mL Basel Consensus thresholds (Luginbühl et al., 2022a) shown in Fig. 2, were positively skewed (skewness = 4.0), as the majority (53.5%) had PEth concentrations below 20 ng/mL. As a matter of fact, 42.2% had PEth concentrations below 10 ng/mL. At the upper end, 10.7% exceeded 200 ng/mL. All unweighted data are provided in supplementary Table S1 and Figures S1-2. Overall, weighting had minimal impact on the distributions.

Given the skewness, the median was used to compare population subgroups. Median estimates with 95% CIs are shown in Table 2. The median PEth concentration in males was 34 ng/mL, which was significantly higher than in females, whose median was <LLoQ (Mann-Whitney test, p < 0.001). An age-dependent trend was observed: the median concentration increased from 11 ng/mL (18–39 years) to 16 and 20 ng/mL in the 40–64 and 65 + groups. Maximum values also increased with age, reaching 430 ng/mL (18–39), 860 ng/mL (40–64), and 1400 ng/mL (65 +). Median PEth concentrations by education, household type and financial hardship were generally below 20 ng/mL or had CIs overlapping this cut-off. Exceptions were “single with children” and a small, unreliable “not specified” financial-hardship group. Dietary, and health variables were not further considered in the analyses due to the defined scope and objectives of the current study. Fig. 2 shows the distribution of PEth concentrations for the entire population and stratifications by sex and age.

### 3.3 Linking PEth concentrations with AUDIT-C scores

**Fig. 3:**
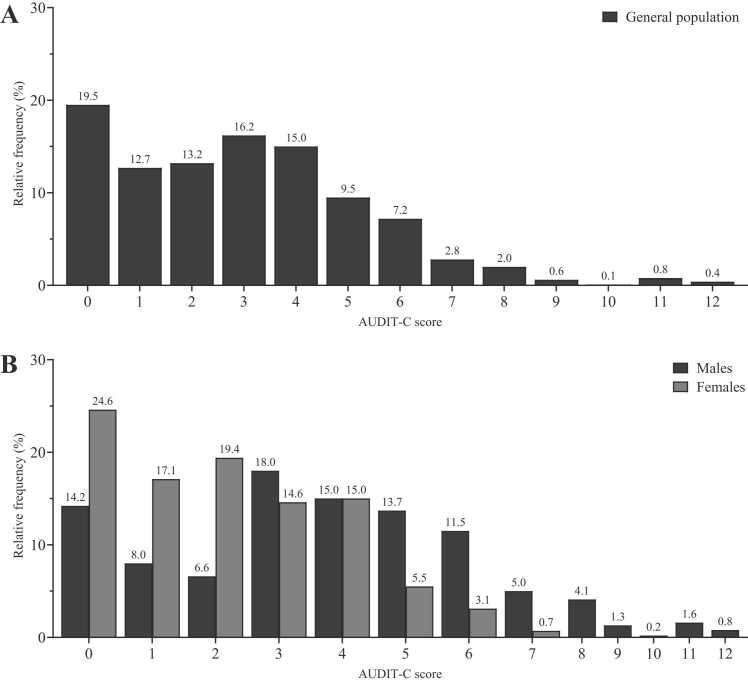
Relative frequency of AUDIT-C scores in the general Belgian adult population. Bars represent the proportion of participants for each AUDIT-C score (0−12), based on population-weighted data (A) and stratified by sex (B).

**Fig. 4:**
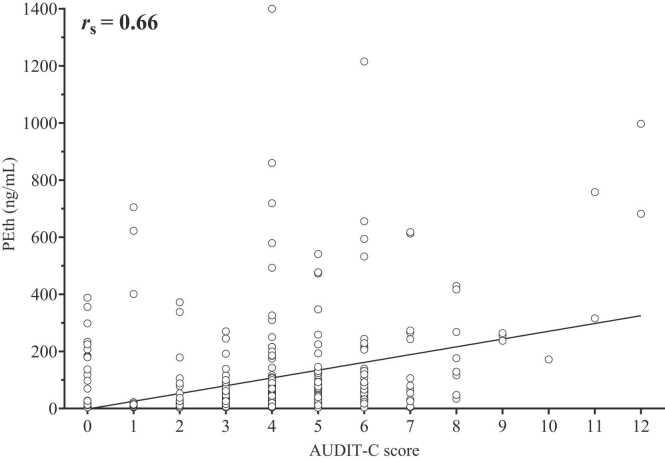
Correlation between participant’s AUDIT-C scores and corresponding PEth concentrations (ng/mL). Spearman’s rank correlation coefficient (*r_s_*) = 0.66.

AUDIT-C scores calculated for the general Belgian population yielded a median score of 3. Because AUDIT-C thresholds differ by sex, results were stratified: females had a median score of 2 (below the hazardous‑drinking threshold), whereas males had a median of 4 (slightly above it) (Fig. 3). The correlation between AUDIT-C and PEth was significant (Spearman *r*_s_ = 0.66, p < 0.001; Fig. 4) with *r*_s_ = 0.61 in males (95% CI: 0.59, 0.63) and 0.64 in females (95% CI: 0.62, 0.66). Unweighted AUDIT-C data are provided in Figure S3.

**Fig. 5:**
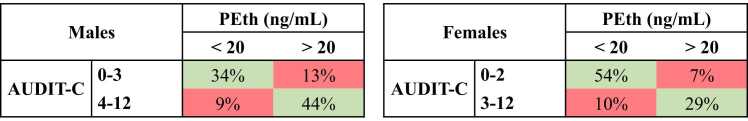
Crosstabs comparing agreement between PEth concentrations to AUDIT-C scores for males and females. Green indicates concordance and red indicates discordance between measures. Analyses are based on n = 242 males and n = 245 females, with results weighted to represent the general Belgian adult population.

PEth concentrations were also cross‑tabulated with AUDIT‑C scores (Fig. 5). Results above the PEth cut-off of 20 ng/mL indicated regular alcohol use; AUDIT-C thresholds AUDIT‑C thresholds for hazardous drinking were ≥ 4 for males and ≥ 3 for females. Concordance between both measures was 78% in males and 83% in females. Among males, 13% had higher PEth relative to AUDIT-C and 9% lower, whereas in females, 7% were higher and 10% lower. Cohen’s Kappa indicated moderate agreement in males (κ = 0.55; 95% CI: 0.52–0.57), and substantial agreement in females (κ = 0.63; 95% CI: 0.61–0.66).

### 3.4 Higher-exposure subgroups

**Fig. 6:**
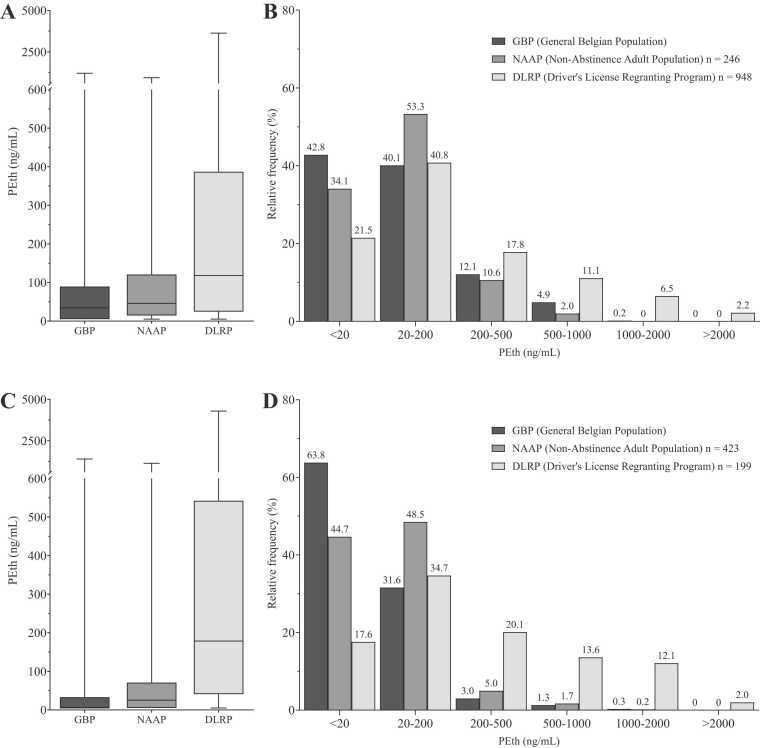
Upper panel: Boxplots (A) and bar chart (B) of PEth concentrations (ng/mL) in males from the general Belgian population (GBP), the non-abstinence adult population (NAAP), and the driver’s license regranting program (DLRP), with median values of 34, 46, and 118 ng/mL, respectively. Lower panel: Boxplots (C) and bar chart (D) of PEth concentrations (ng/mL) in females across the same groups, with median values of <LLoQ (10 ng/mL), 25 ng/mL, and 171 ng/mL, respectively. Quantification range was up to 2000 ng/mL; plotted values exceeding this range were extrapolated.

The results obtained for the general Belgian population were used for a comparative assessment with those obtained for a total of 669 and 1147 individuals in a NAAP (Van Uytfanghe et al., 2022b) and a DLRP, respectively (Fig. 6). The median PEth concentrations in these populations were 32 ng/mL (95% CI: 27, 38; skewness = 4.0) and 122 ng/mL (95% CI: 110, 143), respectively. In the forensic subpopulation, 2% had PEth levels exceeding 2000 ng/mL (skewness = 2.8). Stratified by sex, the respective median concentrations were respectively 46 ng/mL (95% CI: 35, 55) and 118 ng/mL (95% CI: 102, 138) for men (n = 246 and 948) and 25 ng/mL (95% CI: 21, 32) and 171 ng/mL (95% CI: 114, 265) for women (n = 423 and 199).

## 4 Discussion

To our knowledge, this study is among the few epidemiological studies assessing the direct alcohol biomarker PEth in the general population, and the first in Belgium. The context of the BFCS offered the opportunity to set up this nationwide survey. In this study, a single PEth measurement was used to assess momentary PEth concentrations in a population sample, whereas repeated sampling of the same individuals would be preferable for longitudinal monitoring of PEth levels (Van Uytfanghe et al., 2022b). Logistically, the availability of VAMS facilitated finger prick-based whole blood sample collection at the participants’ homes. Although sampling was supervised by trained personnel, sample quality was not always guaranteed. About 75% of the sampling kits received in the lab contained at least one good quality sample, i.e. no under- or overfilling and stored in a zip-lock bag with a silica desiccant. This success rate is in line with previous studies performed using 10 µL Mitra® samplers (Boffel et al., 2024, Van Uytfanghe et al., 2021b). As part of the broader national food consumption study (Heughebaert et al., 2026), two good quality replicates were needed, the first for thiamine determination, and the second for measuring PEth. This requirement for a second good quality sample reduced the ‘success rate’ to ~56%. Additional sample attrition occurred between sampling and analysis, mainly related to administrative (consent withdrawal, incomplete participant data) and technical factors, resulting in 487 samples with valid PEth measurements. The loss of ~44% of samples may have affected the generalisability of the findings. To mitigate this, weighting factors were applied during data analysis to improve the representativeness of the final sample with respect to the Belgian population. It also underscores the importance of clear sampling instructions when implementing dried blood microsampling in larger-scale population studies, in order to maximize the proportion of good-quality samples obtained. Nevertheless, residual selection bias cannot be fully excluded and should be considered when interpreting the results.

The BFCS involved voluntary participation from the general population, regardless of a history of alcohol consumption, thereby including both PWDA and abstainers. In this population, 53.5% had PEth concentrations below the 20 ng/mL cut-off, indicating abstinence or a limited alcohol consumption during the weeks (up to about a month) prior to sampling in more than half of the population. PEth levels were <LLoQ of 10 ng/mL in 42.2%, compatible with complete abstinence or a very low alcohol intake.

In the same cohort, 19.5% of participants reported 12-month abstinence through an AUDIT-C score of 0, which is consistent with findings from the 2013, 2018 and 2023–2024 Belgian national health interview surveys, where respectively 18%, 23% and 22% of the population reported not consuming alcohol in the past 12 months (Gisle et al., 2018, Gisle et al., 2025). However, these self-reported measures and PEth concentrations are not directly comparable, as they reflect different assessment periods and fundamentally different measures of alcohol consumption. Whereas the AUDIT-C evaluates self-reported drinking behaviour over a longer retrospective period, PEth objectively reflects alcohol exposure during approximately the preceding 4–5 weeks, a timeframe in which the frequency of alcohol consumption does not necessarily correspond to that assessed by the longer recall period of the AUDIT-C questionnaire (Luginbühl et al., 2022b, Van Uytfanghe et al., 2022a).

Importantly, combining PEth with self-reported questionnaires provided additional insight into drinking patterns within the BFCS cohort. While self-reported measures remain valuable for assessing drinking behaviour at the population level, PEth identified individuals with biomarker concentrations compatible with excessive alcohol consumption, including some participants whose reported alcohol use did not indicate excessive drinking. This discordance may partly reflect limitations inherent to interview-based surveys, including social desirability bias or recall bias, which can lead to underestimation of actual alcohol consumption. People may fear the negative consequences of disclosing their true alcohol consumption and often pour more than a standard unit (10 g alcohol) when serving drinks themselves. Additionally, inter-individual variability in both PEth formation and elimination may also contribute to discrepancies between biomarker concentrations and self-reported alcohol use (Hill-Kapturczak et al., 2018, Javors et al., 2016, Stenton et al., 2019). Together, these findings illustrate the complementary value of PEth as an objective biomarker alongside questionnaire-based approaches and suggest that excessive alcohol consumption in the general population may be underestimated when relying solely on self-reported survey data.

Most studies assessing PEth are conducted in specific settings in which the biomarker is determined in subpopulations mainly composed of PWDA or in individuals that are specifically monitored for their alcohol use. Examples include assessment of fitness-to-drive, monitoring programs, forensic psychiatry, and diagnosing alcohol-related conditions (Isaksson et al., 2011, Wurst et al., 2015). Therefore, median results reported in those studies are typically higher compared to the observed median in this study. For example, in the study conducted by Walther et al. (2015) PEth was determined in alcohol dependent participants, yielding a median concentration of 0.62 µmol/L (~436 ng/mL). Other studies in populations strongly associated with alcohol consumption have reported moderate to high median PEth levels, including 70.4 ng/mL in people with excessive alcohol consumption (Schröck et al., 2017), 176 ng/mL in organ donors with a history of unhealthy alcohol use abuse (Lowery et al., 2018), and 216 ng/mL in a mixed cohort of patients from medical care and alcohol detoxification units (Afshar et al., 2017). These concentrations are noticeably higher than the median PEth of 17 ng/mL obtained in this study, in which no specific subpopulations were targeted. A large Norwegian population-based study, the HUNT 4 Study, reported a median PEth concentration of < 0.030 µmol/L (~21 ng/mL) among 24 574 participants (Skråstad et al., 2023), aligning with this study’s median of 17 ng/mL. In contrast, a retrospective American study by (Mathewson et al., 2025), revealed that 68% of the 235 504 analyzed specimens had PEth concentrations < 10 ng/mL, an even greater proportion than observed in this study.

Stratification by sex revealed higher median PEth levels in males (34 ng/mL), consistent with alcohol consumption, while over half of females had levels < 10 ng/mL, indicating minimal or no intake. This difference should not be attributed to sex-based variation in PEth metabolism, as previous studies suggest that PEth formation and degradation are not affected by sex, age, ethnicity, or blood collection method (Finanger et al., 2024, Hahn et al., 2021, Hill-Kapturczak et al., 2018, Wurst et al., 2010). Our findings therefore reflect the global tendency toward higher alcohol consumption among males, with heavy episodic drinking also being more prevalent in males (World Health Organization, 2024). The latter can explain the higher frequency of PEth levels between 20 and 200 ng/mL and > 200 ng/mL in males (40% and 17%) compared to females (32% and 5%).

The median PEth concentration in the age group 18–39 years (11 ng/mL) was lower than in the age groups 40–64 and 65 +  years (16 and 20 ng/mL), with overlapping 95% CIs for the latter two. The maximum observed PEth concentrations also increased with age. As PEth formation or metabolism is not affected by age (Hahn et al., 2021), these differences likely reflect higher alcohol consumption in older age groups. Nonetheless, all age group medians remained ≤ 20 ng/mL, indicating generally low consumption. These results align with national data reporting similar alcohol intake across age groups in terms of quantities, with a slightly higher proportion of people who do not drink alcohol under 24 years. However, consumption patterns change with age, as heavy episodic drinking is more prevalent among younger individuals, whereas daily alcohol consumption is more common in older groups (Gisle et al., 2025). This was recently confirmed by Tevik et al. (2025), who reported a significant trend toward less heavy drinking with increasing age in the Norwegian middle-aged population (≥43 years). In contrast to the more frequent daily consumption observed in the Belgian national health survey, PEth levels reported by Tevik et al. suggested a higher rate of abstinence.

PEth has shown strong correlations with self-reported alcohol intake via the AUDIT-C, with Spearman’s coefficients ranging from 0.48 to 0.71 (Francis et al., 2015, Schröck et al., 2017, Van Uytfanghe et al., 2022b, Walther et al., 2015). Similarly, this study found a coefficient of 0.66, which is in close agreement with the 0.71 reported in the 2022 study by Van Uytfanghe et al. (2022b), further emphasizing the biomarker’s validity in the general population. Nonetheless, discordant results were observed in 22% of males and 17% of females. In 9% of males and 10% of females, PEth levels were lower than expected, possibly due to overreporting or reduced intake in the weeks prior to sampling, while underreporting was more common in males (13%) than females (7%). Agreement between PEth concentrations (cut-off of 20 ng/mL) and AUDIT-C scores (cut-offs for hazardous drinking: 4 for males and 3 for females) was also assessed using Cohen’s Kappa, yielding a moderate agreement for males (κ = 0.55) and a substantial agreement for females (κ = 0.63). This suggests that in this cohort, AUDIT-C scores of females align more closely with objectively measured PEth concentrations. Of course, tests like the AUDIT-C can be subject to recall bias and participants may intentionally provide inaccurate answers to minimize personal, professional or legal consequences (Jastrzębska et al., 2016). However, in this study, PEth results had no personal consequences, so in principle there was no incentive to misreport alcohol use, other than providing ‘desirable’ answers. Differences in the time windows captured by PEth and AUDIT-C may also contribute to discordant results, as mentioned earlier. Additionally, other factors such as inter-individual variability in PEth formation and elimination cannot be excluded.

Interestingly, 3% of the population provided AUDIT-C responses that were markedly inconsistent with their PEth levels. Although PEth is typically absent in abstinent individuals (Hartmann et al., 2007, Hasken et al., 2023), 2.3% reported no alcohol use (AUDIT-C = 0) but had PEth > 20 ng/mL, including 1.1% with levels > 200 ng/mL. Another 0.7% had similar PEth levels despite low AUDIT-C scores (1−2). Based on findings from a separate study by Van Uytfanghe and Stove (2023) which reported a 0.98 negative predictive value for PEth < 20 ng/mL after 27 days of abstinence from an initial 200 ng/mL, it is very unlikely that limited drinking could yield such high PEth levels. This supports the conclusion that these AUDIT-C scores are inaccurate.

A clear shift in the distribution of PEth concentrations was observed when comparing the general population to the in-house data of the two subgroups (NAAP & DLRP) with higher alcohol intake. Median PEth concentrations in the NAAP were 32 ng/mL, compared to 17 ng/mL in this study. Among males, medians were 46 ng/mL versus 34 ng/mL, and among females, 25 ng/mL versus < 10 ng/mL, respectively. As the NAAP included both people with moderate and heavy alcohol consumption, a larger proportion had PEth levels between 20 and 200 ng/mL (53% of males, 48% of females), while most individuals in the general population fell below 20 ng/mL (Fig. 6). In contrast, the DLRP showed a substantially higher median of 122 ng/mL. Individuals in this forensic subgroup underwent a fitness-to-drive evaluation, which included PEth determination, after temporarily losing their driving privileges following a positive roadside alcohol and/or drug test. As shown in Fig. 6, 38% of males and 48% of females in this group had PEth levels > 200 ng/mL, indicative of excessive alcohol use, compared to only 17% and 5% in the general population.

In conclusion, the results obtained in this epidemiological survey, conducted in the context of the BFCS, demonstrated the utility of VAMS for assessing PEth in the general Belgian population. More than half of the participants had concentrations below 20 ng/mL, indicating low or no alcohol use, while 11% showed levels consistent with excessive intake. Results confirmed higher alcohol consumption in males and strong correlations between PEth and AUDIT-C scores. The comparison with other population groups exhibiting higher alcohol intake underscores the value of population-based PEth data for contextualizing forensic and clinical cases.

## CRediT authorship contribution statement

**Liesl Heughebaert:** Writing – review & editing, Methodology, Investigation, Conceptualization. **Stove Christophe:** Writing – review & editing, Supervision, Methodology, Conceptualization. **Evy De Boosere:** Writing – review & editing, Investigation. **Nicolas Berger:** Writing – review & editing, Methodology, Formal analysis, Data curation, Conceptualization. **Katleen Van Uytfanghe:** Writing – review & editing, Methodology, Investigation, Conceptualization. **Kevin Vandenbroucke:** Writing – review & editing, Writing – original draft, Visualization, Investigation, Formal analysis, Data curation.

## Informed consent

Informed consent was obtained from all volunteers included in this study.

## Declaration of Generative AI and AI-assisted technologies in the manuscript preparation process

During the preparation of this work the author(s) used ChatGPT in order to enhance the clarity and linguistic quality of the text. After using this tool/service, the author(s) reviewed and edited the content as needed and take(s) full responsibility for the content of the published article.

## Funding sources

Kevin Vandenbroucke would like to thank the 10.13039/501100007229Special Research Fund (BOF) from Ghent University for granting him a PhD fellowship (BOF23/DOC/036). Liesl Heughebaert would like to thank Special Research Fund (BOF) from Ghent University for granting her a PhD fellowship (01D05220). The Belgian Food Consumption was funded by Sciensano, the Flemish Region and Community, the Walloon Region, the Common Community Commission, the French Community Commission, and the German-speaking Community of Belgium.

## Declaration of Competing Interest

The authors declare that they have no known competing financial interests or personal relationships that could have appeared to influence the work reported in this paper.

## Footnotes

Appendix A Supplementary data associated with this article can be found in the online version at doi:10.1016/j.dadr.2026.100451.
