# Supplementary material for: Reference values for the alcohol biomarker phosphatidylethanol (PEth) in the Belgian population: Insights from a nationwide microsampling study
Source: Drug Alcohol Depend Rep. 2026 May 23;20:100451. doi: 10.1016/j.dadr.2026.100451 (PMC13264116; doi:10.1016/j.dadr.2026.100451)
Supplement: Supplementary file 1 — Supplementary material [file mmc1.pdf]

Reference Values for the Alcohol Biomarker Phosphatidylethanol (PEth) in the Belgian Population: Insights from a Nationwide Microsampling Study

Kevin Vandenbroucke[a], Katleen Van Uytfanghe[a], Liesl Heughebaert[a], Nicolas Berger[b], Evy De Boosere[c], and Christophe P. Stove[a*]

[a]Laboratory of Toxicology, Department of Bioanalysis, Faculty of Pharmaceutical Sciences, Ghent, Belgium.

[b]Department of Epidemiology and Public Health, Scientific Institute of Public Health (Sciensano), Brussels, Belgium

[c]Department of Forensic Pathology, Ghent University Hospital, Ghent, Belgium

***Corresponding author:** Ottergemsesteenweg 460, B-9000 Ghent, Belgium; christophe.stove@ugent.be

Table S1:
Unweighted median and maximum PEth concentrations (ng/mL) and 95% confidence intervals for all subgroups.

	Median PEth conc. (95% CI)		Max PEth conc.
**All participants**	18	(13, 24)	1400
**Sex**			
**Male**	35	(27, 45)	1216
**Female**	*<LLoQ*	(*<LLoQ*, 13)	1400
**Age**			
**18-39**	17	(*<LLoQ*, 27)	430
**40-64**	14	(11, 25)	860
**65 and older**	23	(14, 31)	1400
**Education level**			
**Secondary or lower**	18	(10, 27)	1216
**Short-type higher education**	15	(*<LLoQ*, 26)	618
**Long-type higher education**	23	(13, 34)	1400
**Missing**	-	-	87
**Household type**			
**Single**	19	(*<LLoQ*, 31)	1216
**Single with children**	17	(*<LLoQ*, 56)	493
**Couple**	21	(13, 31)	1400
**Couple with children**	17	(12, 26)	705
**Other**	11	(*<LLoQ*, 33)	719
**Ends meet**			
**Very easily**	17	(*<LLoQ*, 31)	618
**Easily**	17	(12, 30)	860
**Rather easily**	26	(13, 37)	1400
**Rather difficultly**	13	(*<LLoQ*, 21)	1215
**Difficultly**	20	(*<LLoQ*, 99)	719
**Very difficultly**	*<LLoQ*	(*<LLoQ,* 259)	682
**Not specified**	23	(*<LLoQ*, 210)	210

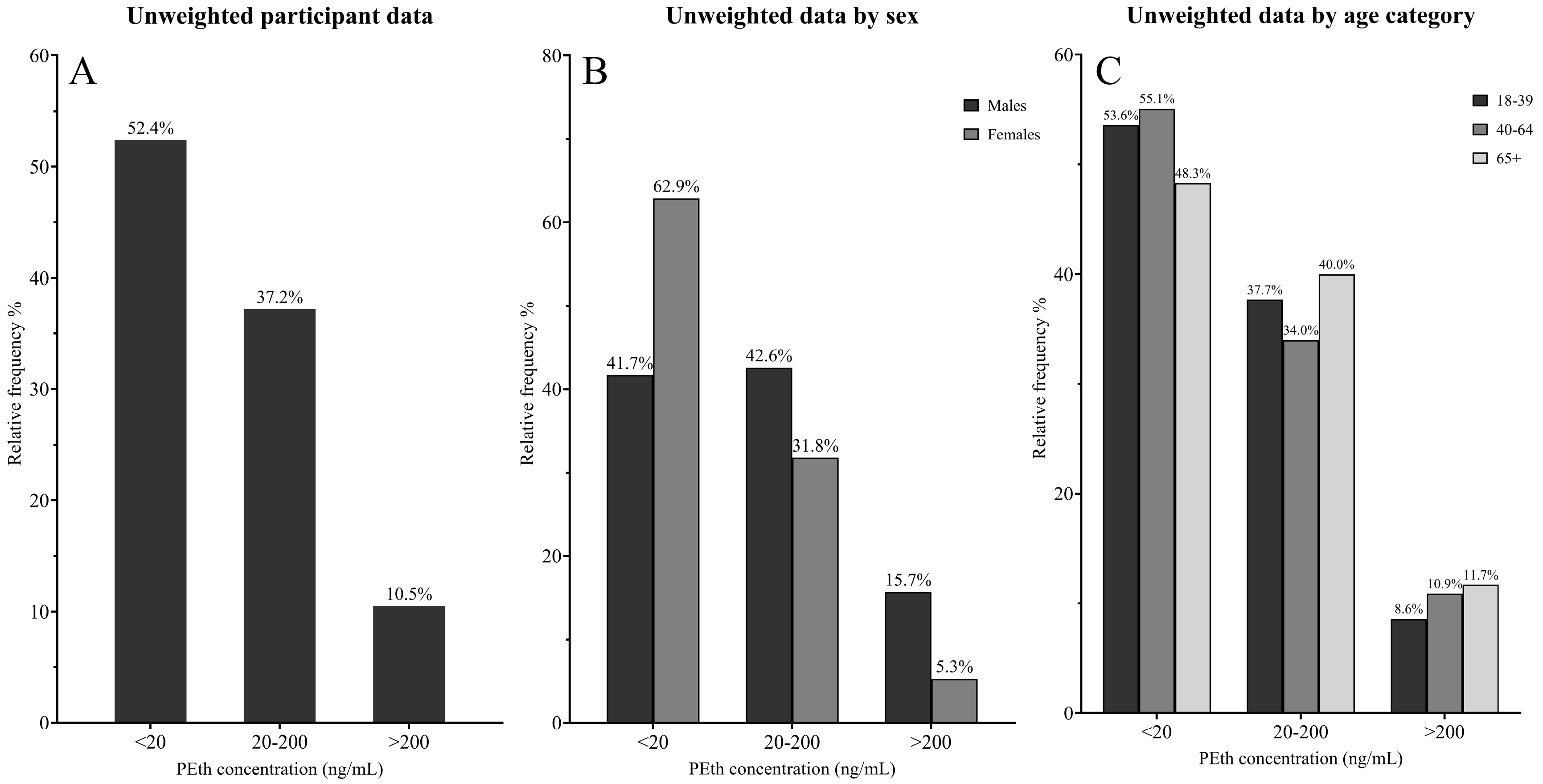

Figure S1: Unweighted distribution of PEth concentrations of the participants (A), stratified by sex (B) and age category (C). Bars represent the relative frequency of participants within three PEth categories (<20 ng/mL, 20-200 ng/mL, >200 ng/mL).

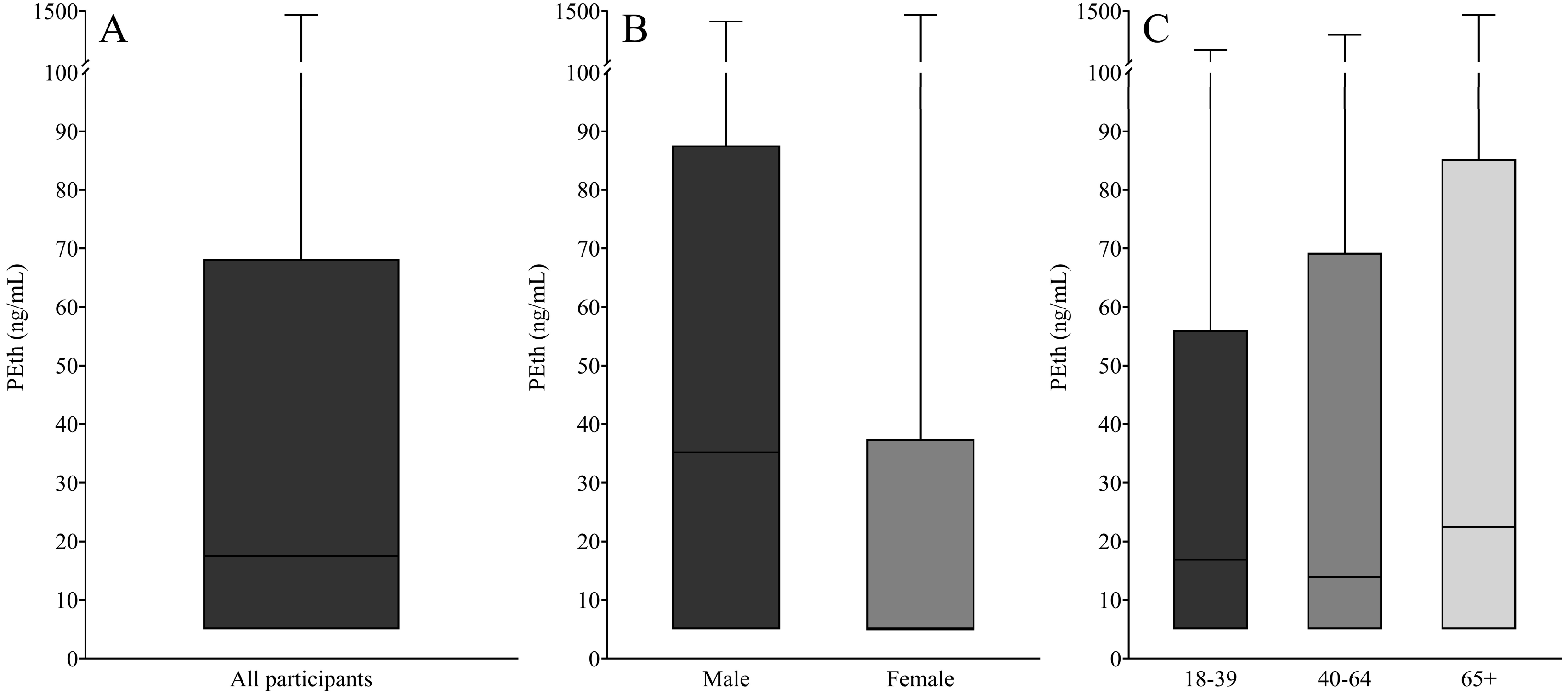

Figure S2: Boxplots (unweighted) showing PEth concentrations (ng/mL) for all participants (A), stratified by sex (B) and age (C). All concentrations <LLoQ of 10 ng/mL were substituted by the value of 5, whiskers indicating minimum-maximum range.

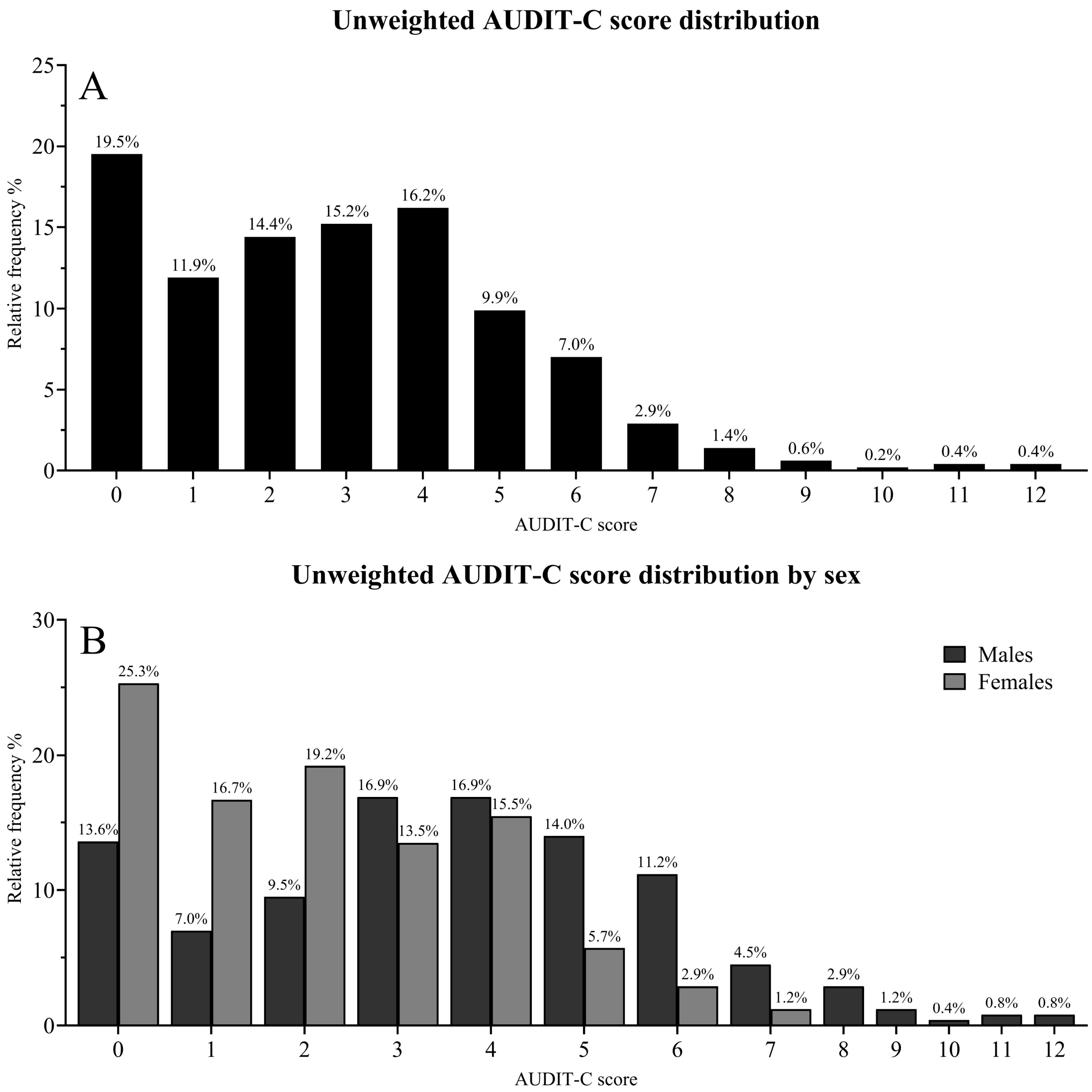

Figure S3: Unweighted relative frequency of AUDIT-C scores for all participants (A) and stratified by sex (B). Bars represent the proportion of participants for each AUDIT-C score (0-12).
